# Sex‐Related Differences in Risk Factors Associated With Nonhealing or Recurrence of Hyperthyroidism in Patients With Graves' Disease Treated With Radioactive Iodine

**DOI:** 10.1002/hcs2.70021

**Published:** 2025-06-13

**Authors:** Haolin Shen, Yuegui Wang, Jianmei Liao, Xianbo Zuo, Bo Zhang, Xiao Yang

**Affiliations:** ^1^ Department of Ultrasound Medicine Zhangzhou Municipal Hospital Affiliated to Fujian Medical University Zhangzhou Fujian China; ^2^ Department of Dermatology China‐Japan Friendship Hospital Beijing China; ^3^ Department of Ultrasound China‐Japan Friendship Hospital, National Center for Respiratory Medicine, National Clinical Research Center for Respiratory Diseases, Institute of Respiratory Medicine of Chinese Academy of Medical Sciences Beijing China; ^4^ Chinese Academy of Medical Sciences & Peking Union Medical College Beijing China; ^5^ Department of Ultrasound Peking Union Medical College Hospital, Chinese Academy of Medical Sciences and Peking Union Medical College Beijing China

## Abstract

**Background:**

To evaluate sex‐related differences in the risk factors associated with nonhealing or recurrence of hyperthyroidism (NHRH) in patients with Graves' disease (GD) treated with radioactive iodine.

**Methods:**

In total, 285 patients were enrolled. Data on radioactive iodine (RAI) dosage, ultrasound indexes of the thyroid, and other clinical factors were collected. Patients were divided into NHRH and non‐NHRH (hypothyroidism or euthyroidism) groups based on treatment outcomes. Univariate and multivariate weighted logistic regression analyses were used to identify factors associated with NHRH. Sex‐specific analyses of these risk factors were also conducted.

**Results:**

There were no significant differences between the two groups in terms of sex, thyroid shear wave elastography velocity values, or pretreatment serum free thyroxine (FT4) levels. Thyroid volume and age were independently associated with NHRH, with the odds of NHRH gradually decreasing as age increased. In subgroup analyses, both age and thyroid volume were independent risk factors for NHRH in female patients (*p* < 0.05), while in male patients, only FT4 was independently associated with NHRH (*p* < 0.05).

**Conclusions:**

In patients of different sexes, the influence of thyroid volume, age, and FT4 on treatment outcomes exhibits distinct patterns.

## Graphical abstract

Age and thyroid volume were independent risk factors for the occurrence of nonhealing or recurrence of hyperthyroidism (NHRH) in female patients. Only free thyroxine (FT4) was independently associated with the occurrence of NHRH in male patients.

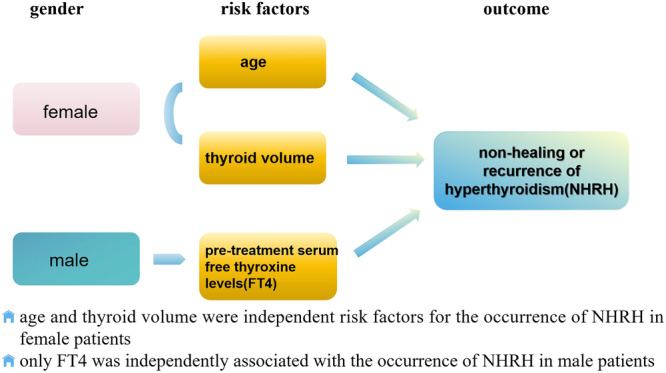

**Abbreviations**
ATD: antithyroid drugs
GD: Graves' disease
NHRH: nonhealing or recurrence of hyperthyroidism
RAI: radioactive iodine
SWE: shear wave elastography

## 1 Introduction

Thyroid dysfunction affects approximately 1.2% of the population, with Graves' disease (GD) accounting for 70%–85% of these cases [1, 2, 3, 4]. GD is characterized by systemic hypermetabolism, diffuse thyroid enlargement, and ocular manifestations [5]. Treatment options for GD include antithyroid drugs (ATDs), thyroidectomy, and radioactive iodine (RAI) therapy [6]. However, managing GD remains challenging because of variable success rates and the potential adverse effects associated with each treatment option.

ATDs are commonly used to treat GD, but their efficacy is limited, with only a 50% success rate after 1 year of treatment [7]. Additionally, ATDs can cause serious adverse effects such as agranulocytosis, aplastic anemia, and liver dysfunction, making long‐term use problematic [8, 9]. Surgical intervention, while effective, carries a high rate of adverse reactions, underscoring the need for alternative treatment approaches [10].

RAI therapy has emerged as a primary treatment for GD in many regions because of its favorable side effect profile and effectiveness in reducing thyroid function [11, 12]. This therapy involves administering a therapeutic dose of iodine‐131 (^131^I), which emits rays that selectively damage thyroid cells, thereby reducing hormone synthesis and secretion [13]. Nonetheless, approximately 8% of patients experience nonhealing or recurrence of hyperthyroidism (NHRH) following RAI therapy, often due to inadequate treatment dosage or individual variations in response to radiotherapy [14, 15].

Understanding the factors influencing treatment outcomes in patients with GD undergoing RAI therapy is crucial for optimizing efficacy and reducing the risk of NHRH. Various demographic and clinical factors, including age, sex, prior use of ATDs, and disease severity, are believed to affect treatment response [16, 17]. However, the specific role of these factors in predicting treatment outcomes and the likelihood of NHRH remains unclear.

Accurate assessment of thyroid volume and characteristics is crucial for determining the appropriate RAI therapy dosage and predicting treatment outcomes [18, 19]. Advanced imaging techniques, such as three‐dimensional ultrasound and ultrasound elastography, provide precise measurements of thyroid volume and hardness. These techniques facilitate informed treatment planning and monitoring, thereby improving the overall management of GD [20, 21].

This study aims to explore sex disparities in the relationship between pretreatment thyroid characteristics and the outcomes of RAI therapy. By elucidating the roles of sex, age, RAI therapy dosage, ultrasound measurements, and free thyroxine (FT4) in treatment response, this study seeks to advance our understanding of personalized management approaches for GD and enhance clinical outcomes for patients undergoing RAI therapy.

## 2 Materials and Methods

### 2.1 Patients

The study involved 329 patients diagnosed with GD who were admitted to the Nuclear Medicine Department of Zhangzhou Municipal Hospital between January 2020 and March 2023. The research concluded on March 1, 2024.

### 2.2 Inclusion and Exclusion Criteria

The inclusion criteria were diagnosis of GD by a clinical physician, absence of contraindications for RAI therapy, and availability of clinical follow‐up data. The exclusion criteria were a history of RAI therapy or thyroid surgery, incomplete pathological data, or < 1 year of follow‐up after RAI therapy without any intervening events by the study deadline.

### 2.3 Definitions of Posttreatment Outcomes

Posttreatment outcomes were categorized based on follow‐up assessments and stratified into two distinct groups. The first category included cases in which symptoms of hyperthyroidism persisted, either worsening over time or showing no improvement, thereby potentially requiring additional cycles of RAI therapy or initiation of antithyroid medication. The second category encompassed cases in which premature hypothyroidism or maintenance of a euthyroid status was observed—defined by subnormal serum FT4 levels with elevated thyroid‐stimulating hormone levels within 1 year following RAI therapy—or in which clinical signs of hyperthyroidism fully resolved, accompanied by the normalization of serum FT4 levels within the same timeframe. Follow‐up for each patient was limited to a maximum of 1 year, beginning 1 month after treatment initiation. Follow‐up ended upon the occurrence of a relevant clinical event, the passage of 1 year, or the conclusion of the study period. Therapeutic efficacy was determined at the discretion of the attending physicians.

### 2.4 Parameters

**Figure 1:**
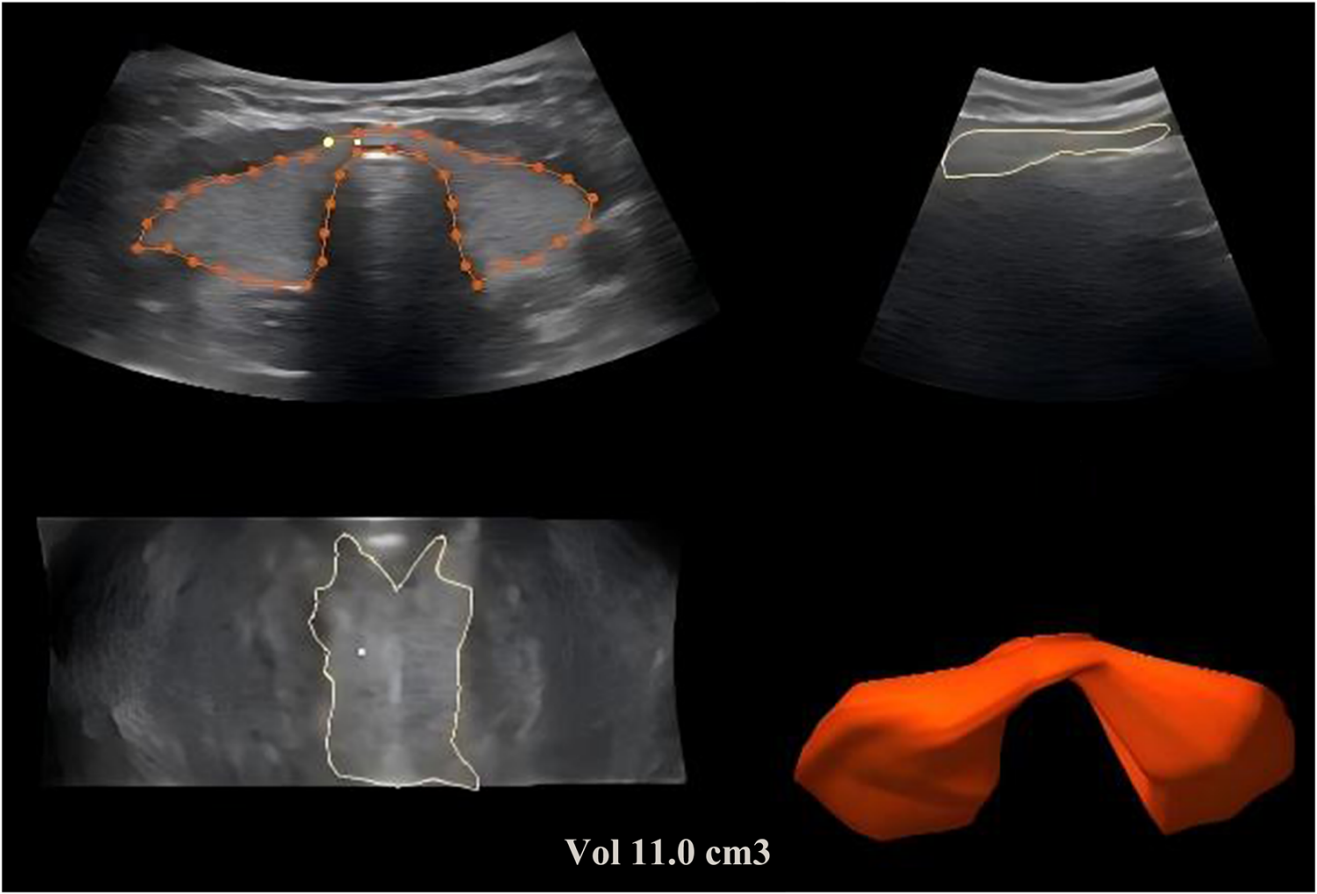
Illustration of normal thyroid three‐dimensional volume measurement.

During each participant's visit to our institution, ultrasound examinations and fasting blood tests were conducted. The ultrasound examination involved three‐dimensional volume measurements using a RAB‐6 probe operating at a frequency range of 7–14 MHz. Measurements were obtained, and computer software automatically generated three‐dimensional images from which thyroid volume was assessed (Figure 1). The standard formula (empirical activity = 70–150 μCi/g of thyroid tissue) was used to calculate the RAI dose based on gland volume and RAI uptake.

Thyroid shear wave elastography (SWE) was performed using a SIEMENS ACUSON Sequoia 512 system (Siemens Medical Solutions USA Inc.) with a 10L4 linear array probe. SWE measurements were taken at the center of the gland every 5 mm, and the average was calculated in elastic imaging mode.

FT4 levels were analyzed using an electroluminescent immunoassay (DXI800; Beckman), with a reference range of 7.5–21.1 pmol/L. Age and sex information was retrieved from the clinical records.

### 2.5 Statistical Analysis

Statistical analyses were performed using *R* Commander, version 4.3.1, available at http://www.r-project.org/. Mean value interpolation was employed to manage missing data. Continuous variables were expressed as mean ± standard deviation for normally distributed data or as median [Q25, Q75] for non‐normally distributed data. Categorical variables were presented as counts (*n*) and percentages (%).

Logistic regression analyses were conducted using the *rms* package. Based on previous research, three different models were established using univariate and multivariate weighted logistic regression to examine the relationship between thyroid volume and treatment outcomes across all male and female participants. The models were defined as follows: unadjusted model (no covariate adjustment), minimally adjusted model (Model I: adjusted for age and sex), and fully adjusted model (Model II: adjusted for sex, age, ^131^I treatment dosage, thyroid SWE velocity values, and pretreatment FT4 levels). In the male and female subgroups, the covariates in Models I and II did not include sex.

## 3 Results

### 3.1 Patients' Demographics and Outcomes

Of the initially selected 329 patients with GD, 30 were excluded because of loss before follow‐up, and 14 had not experienced relevant events before the study deadline. Consequently, 285 patients (mean age, 43.0 ± 14.2 years) were included in the analysis. There were no significant differences in baseline characteristics, such as age and sex, between excluded and included patients.

Among the included participants, 87 experienced NHRH, while 198 developed premature hypothyroidism or maintained a euthyroid status (non‐NHRH group).

### 3.2 Baseline Characteristics of the Study Participants

The patients' demographic and clinical characteristics showed no significant differences between the two groups in terms of sex, thyroid SWE velocity values, and pretreatment serum FT4 levels (all *p* > 0.05). However, significant differences were observed in treatment dosage, age, and thyroid volume (all *p* < 0.05) (Table 1).

**Table 1:** Baseline characteristics of the study participants.

	Hypothyroidism or euthyroidism	NHRH	
	*N* = 198	*N* = 87	*p* overall
Sex			0.247
Female	149 (75.3%)	59 (67.8%)	
Male	49 (24.7%)	28 (32.2%)	
Age			0.006
< 30	28 (14.1%)	26 (29.9%)	
30–60	144 (72.7%)	54 (62.1%)	
> 60	26 (13.1%)	7 (8.0%)	
Dose	6.5 [5.1; 8.4]	7.7 [5.8; 10.3]	0.006
Volume			< 0.001
Normal	88 (44.4%)	16 (18.4%)	
Mild enlargement	95 (48.0%)	47 (54.0%)	
Severe enlargement	15 (7.6%)	24 (27.6%)	
SWE	1.7 [1.5; 2.0]	1.7 [1.5; 2.1]	0.999
FT4	37.0 [23.0; 56.2]	36.3 [23.1; 51.1]	0.849

Abbreviations: FT4, serum free thyroxine; NHRH, nonhealing or recurrence of hyperthyroidism; SWE, shear wave elastography.

### 3.3 Results of Univariate Analysis and Multivariable Weighted Logistic Regression Models

**Figure 2:**
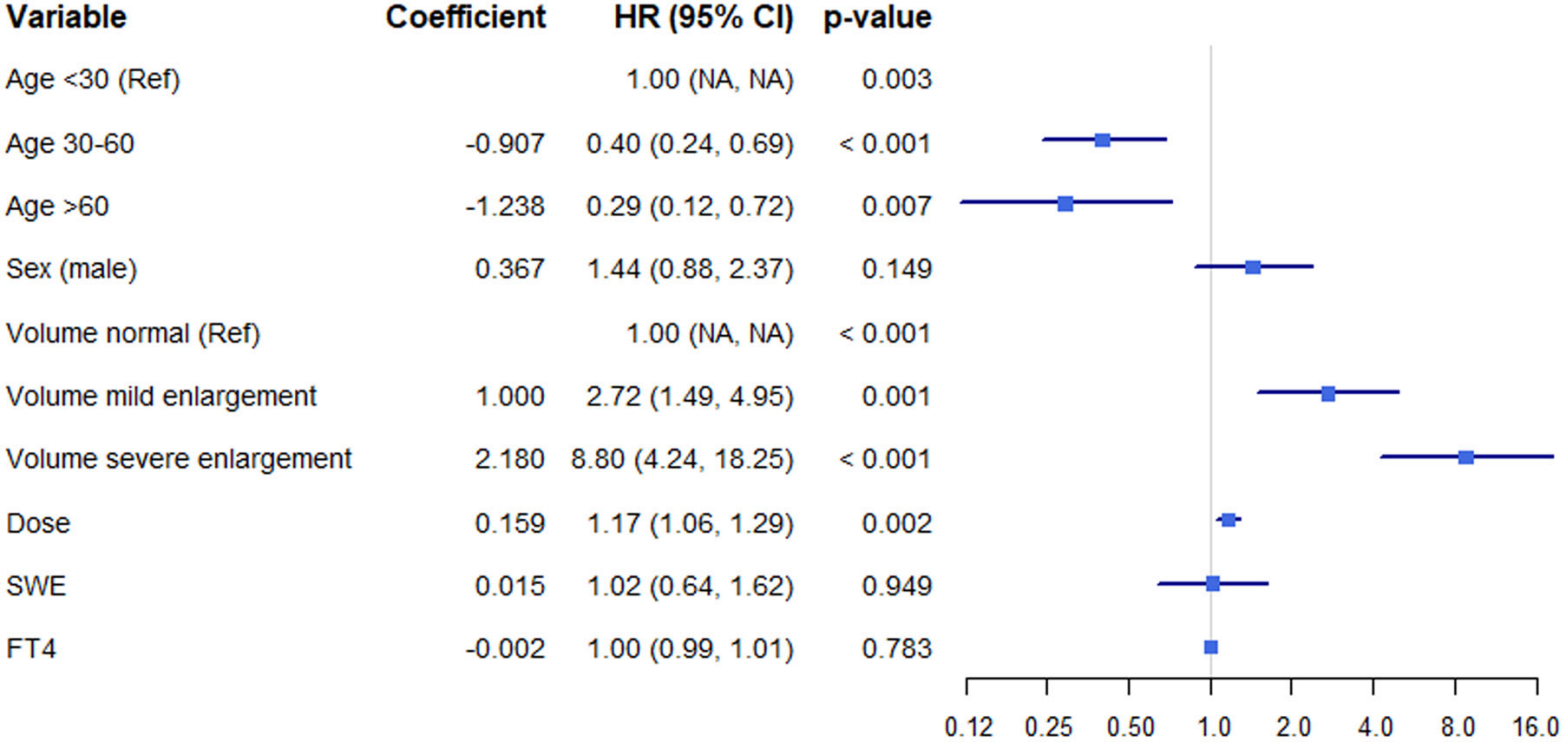
Association between clinical factors and NHRH in the univariate analysis. FT4, serum free thyroxine; NHRH, nonhealing or recurrence of hyperthyroidism; SWE, shear wave elastography

Univariate and multivariable weighted logistic regression analyses were conducted (Figure 2 and Table 2). Model 1 was adjusted for age, sex, and thyroid volume, while Model 2 additionally included adjustments for treatment dosage, SWE, and FT4 levels. The results indicate that thyroid volume and age are independently associated with NHRH (*p* < 0.001).

**Table 2:** Results of univariate analysis and multivariable weighted logistic regression model.

	Univariate analysis	Model I	Model II
	(OR, 95% CI); *p*‐value	(OR, 95% CI); *p*‐value	(OR, 95% CI); *p*‐value
Sex			
Female	Ref	Ref	Ref
Male	1.44 (0.88–2.37); 0.149	1.13 (0.66–1.93); 0.658	1.14 (0.66–1.96); 0.647
Age		0.061	0.055
< 30	Ref	Ref	Ref
30–60	0.4 (0.24–0.69); < 0.001	0.55 (0.31–0.98); 0.042	0.54 (0.3–0.97); 0.038
> 60	0.29 (0.12–0.72); 0.007	0.37 (0.15–0.96); 0.040	0.36 (0.14–0.94); 0.036
Dose	1.17 (1.06–1.29); 0.002	—	1.04 (0.93–1.17); 0.498
Volume	< 0.001	—	< 0.001
Normal	Ref	Ref	Ref
Mild enlargement	2.72 (1.49–4.95); 0.001	2.51 (1.36–4.61); 0.003	2.86 (1.5–5.48); 0.002
Severe enlargement	8.80 (4.24–18.25); < 0.001	7.28 (3.38–15.7); < 0.001	8.5 (3.45–20.95); < 0.001
SWE	1.02 (0.64–1.62); 0.949	—	0.74 (0.43–1.25); 0.254
FT4	1.00 (0.99–1.01); 0.781	—	0.99 (0.97–1.00); 0.062

Abbreviations: CI, confidence interval; FT4, serum free thyroxine; OR, odds ratio; SWE, shear wave elastography.

### 3.4 Subgroup Analysis According to Sex

In female patients, age and thyroid volume were independent risk factors for the occurrence of NHRH (both *p* < 0.05), while in male patients, only FT4 was independently associated with NHRH (*p* < 0.05) (Table 3).

**Table 3:** Independent risk factors for NHRH, stratified by Sex.

	Female		Male	
	Univariate analysis	Multivariate analysis	Univariate analysis	Multivariate analysis
Age				
< 30	Ref	Ref	Ref	
30–60	0.001	0.138	0.271	—
> 60	0.012	0.030	0.285	—
Dose	0.040	0.855	0.023	0.056
Volume				
Normal	Ref	Ref	Ref	Ref
Mild enlargement	0.001	0.002	0.468	0.649
Severe enlargement	< 0.001	< 0.001	0.026	0.151
SWE	0.478	—	0.574	—
FT4	0.266	—	0.039	0.005

Abbreviations: FT4, serum free thyroxine; NHRH, nonhealing or recurrence of hyperthyroidism; SWE, shear wave elastography.

## 4 Discussion

In previous studies, our cumulative risk margin probability analysis showed that the probability of NHRH 300 days after RAI treatment was approximately 25% [22]. This indicates a significant rate of treatment failure and underscores the need for a deeper understanding of the factors influencing outcomes, as well as the implementation of targeted measures to improve success rates. Prior research has primarily examined factors such as age, sex, duration of GD, history of ATD use before RAI treatment, RAI dosage and half‐life, and pretreatment thyroid‐stimulating hormone receptor antibody positivity. However, findings across studies have varied considerably [23, 24].

The results of this study further confirm that a larger thyroid volume is associated with a higher risk of NHRH. A larger thyroid volume may reflect a greater disease burden and heightened autoimmune activity within the thyroid tissue, both of which contribute to the increased risk of NHRH following RAI treatment. These findings are consistent with earlier research and suggest that thyroid volume should be carefully considered when planning treatment [19]. For patients with larger thyroid volumes, more personalized treatment strategies may be necessary to improve efficacy and reduce the risk of treatment failure.

This study found that when analyzing patient age as a continuous variable, there was no significant correlation between age and NHRH (*p* > 0.05). However, when patients were divided into three subgroups based on age thresholds of 30 and 60 years, significant differences in NHRH incidence were observed among the groups (*p* < 0.05). This suggests that the relationship between age and treatment outcome is nonlinear. As age increases, the overall risk of NHRH tends to decrease across the patient population. This trend may be associated with age‐related physiological changes, including alterations in immune function, hormone levels, and thyroid activity. Additionally, the impact of age on treatment outcomes appears to differ by sex. While female patients show a declining trend in NHRH risk with increasing age, the influence of age on treatment outcomes in male patients is not significant.

Previous study has indicated that females are more prone to experiencing NHRH [25], and this study further confirms that factors associated with treatment outcomes vary significantly between sexes. Several factors may contribute to this difference, including genetic influences, variations in sex hormones, immune system responses, and differing organ susceptibility [26, 27, 28]. A detailed sex‐stratified analysis reveals that the factors influencing treatment outcomes are not the same for males and females. In female patients, the risk of NHRH increases proportionally with thyroid volume and age. Conversely, in male patients, adverse treatment outcomes are significantly associated with FT4 levels. These sex‐based differences in the impact of thyroid volume on treatment outcomes may stem from underlying biological distinctions, such as differences in hormone levels and immune responses. Studies have shown that thyroid peroxidase antibody levels in females are 2–5 times higher than in males, partly explaining the stronger association between larger thyroid volumes and treatment efficacy in female hyperthyroid patients [29]. These findings suggest that sex differences should be considered when devising treatment plans. For patients with larger thyroid volumes—especially females—higher doses of RAI therapy or alternative treatment strategies may be necessary to optimize outcomes. While male patients with mild thyroid volume enlargement may not exhibit markedly different treatment responses, caution is still warranted in cases of severe enlargement due to the risk of treatment failure.

Sex‐related differences should be fully considered when developing treatment plans, particularly for patients with larger thyroid volumes. Because of distinct immune response characteristics and higher thyroid peroxidase antibody levels in female patients, higher doses of RAI therapy may be necessary, or alternative treatment options—such as surgery or antithyroid medications—should be considered. In male patients, although the relationship between thyroid volume and treatment response is less pronounced, the risk of treatment failure in cases of severe thyroid enlargement should still be closely monitored, especially given the influence of FT4 levels on treatment outcomes.

These findings have practical implications for clinicians, emphasizing the importance of considering age when devising treatment plans. Older female patients may be expected to have better treatment outcomes, while younger patients or male patients may require closer monitoring for treatment efficacy and risk of recurrence. Limitations of this study include the relatively small sample size, single‐center design, and lack of long‐term follow‐up data. In future research, we aim to expand the sample size and conduct multicenter studies to further validate sex differences in the impact of relevant factors on treatment outcomes.

## 5 Conclusions

The impact of increasing thyroid volume and age on treatment outcomes—specifically, non‐resolution or recurrence of hyperthyroidism—differs between sexes. Therefore, it is essential to consider sex‐specific differences when formulating treatment plans.

## Author Contributions

**Haolin Shen:** formal analysis (lead), funding acquisition (lead), project administration (lead), writing – original draft (lead). **Yuegui Wang:** data curation (lead), formal analysis (equal), writing – original draft (equal). **Jianmei Liao:** data curation (equal), resources (equal). **Xianbo Zuo:** writing – review and editing (equal). **Bo Zhang:** writing – eview and editing (equal). **Xiao Yang:** supervision (lead), writing – review and editing (equal).

## Ethics Statement

This study received ethical approval from the research ethics committee of Zhangzhou Municipal Hospital, affiliated with Fujian Medical University (Approval No. 2020KYB138).

## Consent

All participants provided written informed consent.

## Conflicts of Interest

Xianbo Zuo is a member of the *Health Care Science* Editorial Board. To minimize bias, he was excluded from all editorial decision‐making related to the acceptance of this article for publication. The remaining authors declare no conflicts of interest.

## Data Availability

The data that support the findings of this study are available on request from the corresponding author. The data are not publicly available due to privacy or ethical restrictions.
